# Supplementary material for: Global Burden Related to Nitrous Oxide Exposure in Medical and Recreational Settings: A Systematic Review and Individual Patient Data Meta-Analysis
Source: J Clin Med. 2019 Apr 23;8(4):551. doi: 10.3390/jcm8040551 (PMC6518054; doi:10.3390/jcm8040551)
Supplement: Supplementary file 1 [file jcm-08-00551-s001.pdf]

Journal of Clinical Medicine

MDPI

Table S1. Detail of the Twenty-Nine Studies Excluded from the Systematic Review

First author, year	Reason of exclusion from the systematic review	Reference
Adornato BT. 1978	Review paper	[1]
Burman JF, Amess JA, et al. 1978	Review paper	[2]
Nunn JF, Chanarin I. 1978	Review paper	[3]
Chanarin I. 1979	Review paper	[4]
Chanarin I. 1980	Review paper	[5]
Nevins MA. 1980	No biological data available	[6]
Gillman MA. 1982	Review paper	[7]
Lunsford JM, Wynn MH. 1983	Congress abstract	[8]
Koblin DD, Biebuyck JF. 1986	Narrative review	[9]
Ueland PM.1987	Not in English	[10]
Rupreht J, Erdmann W, *et al.* 1989	Not in English	[11]
Ostreicher DS. 1994	Review paper	[12]
Hoerauf K, Koller C, *et al.* 1995	Not in English	[13]
King M, Coulter C, *et al.* 1995	No available data	[14]
Jongen JC, Koehler PJ, *et al.* 1995	Not in English	[15]
Nestor PJ, Stark RJ. 1996	No biological data available	[16]
Takacs J.1996	Not in English	[17]
Lai NY, Silbert PL. 1997	Congress abstract	[18]
Mayall M. 1999	Review paper	[19]
Alarcia R, Ara JR, *et al.* 1999	Not in English	[20]
Gothe CJ, Petersson G.1999	Not in English	[21]
Lindstedt G. 1999	Not in English	[22]
Barbosa L, Leal I, *et al.* 2000	Not in English	[23]
Vinciguerra C, Chazerain P, *et al.* 2000	Not in English	[24]
Smith I. 2001	Review paper	[25]
Deleu D, Hanssens Y, et al. 2001	Review paper	[26]
England JM, Linnell JC. 2001	Review paper	[27]
Van Geffen GJ, de Boer HD, *et al.* 2001	Not in English	[28]
Burman JF. 2003	Editorial	[29]
Erbe RW, Salis RJ. 2003	Narrative review	[30]
Yamada T, Hamada H. 2005	Subject not exposed to N_2O	[31]
Gerges FJ, Dalal AR. 2006	Previously treated with vitamin B12	[32]
Cohen Aubart F, Sedel F *et al.* 2007	Not in English	[33]
El Otmani H, El Moutawakil B, *et al.* 2007	Not in English	[34]
Luzardo GE, Karlnoski RA, et al. 2008	Review paper	[35]
El Otmani H, El Moutawakil B, *et al.* 2009	Not in English	[36]
Paul I, Reichard RR. 2009	Subject not exposed to N_2O	[37]

**Asghar A, Ali FM. 2012**	Previously treated with vitamin B12	[38]
**Chaugny C, Simon J, *et al.* 2014**	Not in English	[39]
**Chin J, Forzani B, et al. 2015**	Review paper	[40]
**Liakoni E, Liechti ME. 2015**	Not in English	[41]
**Goodman BP. 2015**	Review paper	[42]
**van Amsterdam J, Nabben T, et al. 2015**	Review paper	[43]
**Garakani A, Jaffe RJ, et al. 2016**	Review paper	[44]
**Ingelmo P, Wei A, *et al.* 2017**	Previously treated with vitamin B12	[45]
**Fluegge K. 2017**	Review paper	[46]
**Stockton L, Simonsen C, et al. 2017**	Review paper	[47]

2

Table S2. Detail of the Studies Retained in the Systematic Review

First author, year	Reference
**Layzer, 1978**	[48]
**Layzer, 1978**	[49]
**Sahenk, 1978**	[50]
**Gutmann**	[51]
**Paulson, 1979**	[52]
**Nunn, 1982**	[53]
**Blanco, 1983**	[54]
**Murdoch, 1985**	[55]
**Heyer, 1986**	[56]
**Schilling, 1986**	[57]
**Berger, 1988**	[58]
**Stabler, 1991**	[59]
**Vishnubhakat, 1991**	[60]
**Stacy, 1992**	[61]
**Hadzic, 1995**	[62]
**Kinsella, 1995**	[63]
**McMorrow, 1995**	[64]
**Rosener, 1996**	[65]
**Brett, 1997**	[66]
**Beltramello, 1998**	[67]
**Pema, 1998**	[68]
**Jameson, 1999**	[69]
**Lee, 1999**	[70]
**Ogundipe, 1999**	[71]
**Butzkueven, 2000**	[72]
**Sesso, 1999**	[73]
**Felmet, 2000**	[74]
**Marie, 2000**	[75]
**McNeely, 2000**	[76]
**Iwata, 2001**	[77]
**Ilniczky, 2002**	[78]
**Ng, 2002**	[79]
**Selzer, 2003**	[80]
**Waclawik, 2003**	[81]
**Diamond, 2004**	[82]
**Doran, 2004**	[83]
**Miller, 2004**	[84]
**Ahn, 2005**	[85]

**Waters, 2005**	[86]
**Sethi, 2006**	[87]
**Cartner, 2007**	[88]
**Levine, 2007**	[89]
**Shulman, 2007**	[90]
**Wu, 2007**	[91]
**Meyers, 2008**	[92]
**Singer, 2008**	[93]
**Huang, 2009**	[94]
**Renard, 2009**	[95]
**Wijesekera, 2009**	[96]
**Richardson, 2010**	[97]
**Tatum, 2010**	[98]
**Alt, 2011**	[99]
**Hathout, 2011**	[100]
**Lin, 2011**	[101]
**Probasco, 2011**	[102]
**Ghobrial, 2012**	[103]
**Hsu, 2012**	[104]
**Sotirchos, 2012**	[105]
**Cheng, 2013**	[106]
**Chiang, 2013**	[107]
**Gursoy, 2013**	[108]
**Safari, 2013**	[109]
**Arshi, 2014**	[110]
**Dababneh, 2014**	[111]
**Garakani, 2014**	[112]
**Hu, 2014**	[113]
**Rheinboldt, 2014**	[114]
**Shwe, 2014**	[115]
**Duque, 2015**	[116]
**Morris, 2015**	[117]
**Pugliese, 2015**	[118]
**Thompson, 2015**	[119]
**Wolpert, 2015**	[120]
**Chen, 2016**	[121]
**Hirvioja, 2016**	[122]
**Massey, 2016**	[123]
**Sleeman, 2016**	[124]
**Buizert, 2017**	[125]
**Kaski, 2017**	[126]
**Yuan, 2017**	[127]

**Chen, 2018**	[128]
**Egan, 2018**	[129]
**Johnson, 2018**	[130]
**Keddie, 2018**	[131]
**Middleton, 2018**	[132]

6 **Table S3.** Variables Associated with Regular Nitrous Oxide Exposure in Univariate Analyses

	Short exposure to N_2O			Regular exposure to N_2O			
	**N**	**Median**	**25–75 P**	**N**	**Median**	**25–75 P**	***p*-value***
**Age (years)**	**24**	**47**	**25–58**	**76**	**26**	**22–33**	**0.002**
**Laboratory findings**							
**Vitamin B12 (pmol/L)**	**21**	**74**	**33–104**	**61**	**110**	**81–194**	**0.0008**
**Folate (serum) (µg/L)**	9	13.2	7.0–16.1	11	12.7	7.3–14.0	0.68
**Hematocrit (%)**	5	34.0	26.8–38.5	16	39.3	34.6–44.2	0.23
**Hemoglobin (g/dL)**	11	11.2	9.5–12.3	32	12.2	8.9–13.8	0.44
**Homocysteine (µmol/L)**	5	111.0	76.4–142.8	26	46.7	28.2–106.0	0.06
**Mean corpuscular volume (fL)**	**15**	**104**	**101–110**	**40**	**97**	**92–101**	**0.0002**
**Methylmalonic acid (µmol/L)**	0	—	—	16	5.0	1.1–6.6	—
**Clinical findings**	**N**	**Percentage**	**95% CI**	**N**	**Percentage**	**95% CI**	***p*-value†**
**Abdominal pain**	24	0.0	—	76	4.0	0–8.4	0.99
**Agitation**	24	4.2	0–12.8	76	1.3	0–3.9	0.42
**Anorexia**	24	4.2	0–12.8	76	0.0	—	0.24
**Apnea**	24	4.2	0–12.8	76	0.0	—	0.24
**Ataxia**	24	8.3	0–20.3	76	13.2	5.4–20.9	0.73
**Athetoid movement**	24	4.2	0–12.8	76	0.0	—	0.24
**Behavior alteration**	24	0.0	—	76	7.9	1.7–14.1	0.33
**Bulbar paralysis**	24	0.0	—	76	1.3	0–3.9	0.99
**Chest pain**	24	0.0	—	76	1.3	0–3.9	0.99
**Mental confusion**	24	4.2	0–12.8	76	2.6	0–6.3	0.56
**Constipation**	24	0.0	—	76	4.0	0–8.4	0.99

**Decreased libido**	24	0.0	—	76	2.6	0–6.3	0.99
**Depression**	24	4.2	0–12.8	76	4.0	0–8.4	0.99
**Disorientation**	24	4.2	0–12.8	76	0.0	—	0.24
**Iterative fallings or equilibrium disorders**	24	12.5	0–26.8	76	27.6	17.3–37.9	0.17
**Fecal incontinence**	24	4.2	0–12.8	76	1.3	0–3.9	0.42
**Foot pain**	24	0.0	—	76	4.0	0–8.4	0.99
**Cognitive decline**	24	12.5	0–26.8	76	7.9	1.7–14.1	0.44
**Headache**	24	4.2	0–12.8	76	4.0	0–8.4	0.99
**Hyperpigmentation**	24	0.0	—	76	4.0	0–8.4	0.99
**Hypotonia**	24	4.2	0–12.8	76	0.0	—	0.24
**Impaired memory**	24	4.2	0–12.8	76	5.3	0.1–10.4	0.99
**Lethargy**	24	8.3	0–20.3	76	0.0	—	0.06
**Lhermitte's sign**	24	8.3	0–20.3	76	17.1	8.4–25.8	0.51
**Neck pain**	24	0.0	—	76	1.3	0–3.9	0.99
**Painful erection**	24	0.0	—	76	1.3	0–3.9	0.99
**Paranoid behavior**	24	0.0	—	76	4.0	0–8.4	0.99
**Paraplegia**	24	4.2	0–12.8	76	0.0	—	0.24
**Paresthesia in extremities, numbness, tingling**	24	70.8	51.2–90.4	76	82.9	74.2–91.6	0.24
**Polyneuropathy**	24	0.0	—	76	1.3	0–3.9	0.99
**Quadriparesis or paralysis**	24	8.3	0–20.3	76	6.6	0.9–12.3	0.67
**Respiratory paralysis**	24	0.0	—	76	1.3	0–3.9	0.99
**Seizures**	24	8.3	0–20.3	76	0.0	—	0.06
**Spasm**	24	0.0	—	76	1.3	0–3.9	0.99
**Suicidal thought**	24	0.0	—	76	1.3	0–3.9	0.99
**Syncope**	24	0.0	—	76	1.3	0–3.9	0.99

**Tachypnea**	24	4.2	0–12.8	76	0.0	—	0.24
**Unsteady gait, walking difficulty**	24	54.2	32.7–75.7	76	59.2	47.9–70.5	0.81
**Urinary incontinence**	24	12.5	0–26.8	76	6.6	0.9–12.3	0.39
**Urine retention**	24	4.2	0–12.8	76	5.3	0.1–10.4	0.99
**Vertigo**	24	0.0	—	76	1.3	0–3.9	0.99
**Visual hallucination**	24	4.2	0–12.8	76	1.3	0–3.9	0.42
**Vomiting**	24	0.0	—	76	1.3	0–3.9	0.99
**Weakness**	24	25.0	6.3–43.7	76	48.7	37.2–60.2	0.06
**Reported diagnoses**							
**Axonal polyneuropathy**	24	12.5	0–26.8	76	10.5	3.5–17.6	0.72
**Vitamin B12 deficiency**	24	25.0	6.3–43.7	76	10.5	3.5–17.6	0.09
**Encephalopathy**	24	4.2	0–12.8	76	1.3	0–3.9	0.42
**Generalized demyelinating polyneuropathy**	24	20.8	3.3–38.4	76	23.7	13.9–33.5	0.99
**MTHFR deficiency**	24	4.2	0–12.8	76	0.0	—	0.24
**Myelopathy**	24	16.7	0.6–32.7	76	28.9	18.5–39.4	0.29
**Recurrent paraparesis**	24	0.0	—	76	1.3	0–3.9	0.99
**Subacute combined degeneration**	24	37.5	16.6–58.4	76	25.0	15–35	0.29
**Toxicity due to N_2O with no specific diagnosis applied**	24	8.3	0–20.3	76	22.4	12.8–32	0.15
**Male gender**	24	58.3	37.1–79.6	76	60.5	49.3–71.8	0.99
**Presence of T2 signal hyperintensity in the spinal cord**	16	81.3	59.8–100	59	64.4	51.8–77	0.24

IQR: interquartile range; **N**: total number of studied patients.
* Mann-Whitney *U* test
† Fisher’s exact test

Journal of Clinical Medicine MDPI

Table S4. Association Between the Amount of Nitrous Oxide (N_2O) Exposure and Main Clinical, Laboratory, and Magnetic Resonance Findings of the N_2O-Related Disease

Main clinical findings	*p*-value*
**Paresthesia in extremities, numbness, tingling**	0.53
**Unsteady gait, walking difficulty**	0.28
**Weakness**	0.55
**Iterative fallings or equilibrium disorders**	0.15
**Ataxia**	0.30
**Laboratory findings**	***p*-value†**
**Hemoglobin (g/dL)**	Low sample size
**Hematocrit (%)**	Low sample size
**Mean corpuscular volume (fL)**	Low sample size
**Vitamin B12 (pmol/L)**	0.08
**Folate (serum) (µg/L)**	Low sample size
**Homocysteine (µmol/L)**	Low sample size
**Methylmalonic acid (µmol/L)**	Low sample size
**Main reported diagnoses**	***p*-value***
**Subacute combined degeneration**	0.36
**Generalized demyelinating polyneuropathy**	0.31
**Myelopathy**	0.91
**Magnetic resonance imaging findings**	***p*-value***
**Presence of T2 signal hyperintensity in the spinal cord**	0.17

* Mann Whitney *U* test

† Spearman's rank correlation coefficient

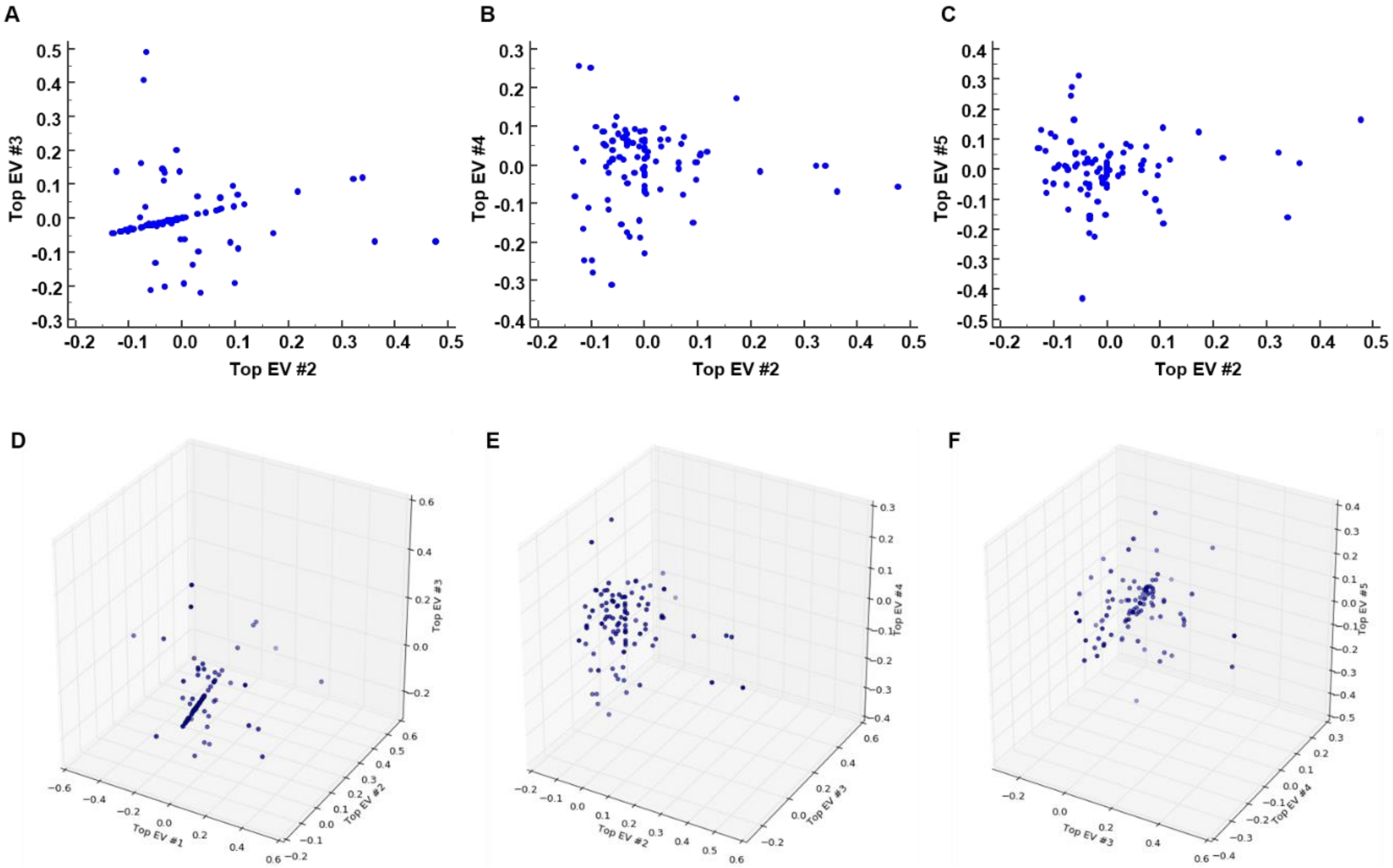

Figure S1. Assessment of study bias induced by population stratification using principal component analysis. Panels (a) to (c) report the two-dimensional visualization of the whole population according to the principal components of the eigenvalue (EV) #2 vs. EV #3 (a), EV #2 vs. EV #4 (b) and EV #2 vs. EV #5 (c). Panels (d) to (f) report the three-dimensional visualization of the whole population according to the principal components for the EV #1, EV #2, and EV #3 (d), EV #2, EV #3, and EV #4 (e), and EV #3, EV #4, and EV #5 (f).

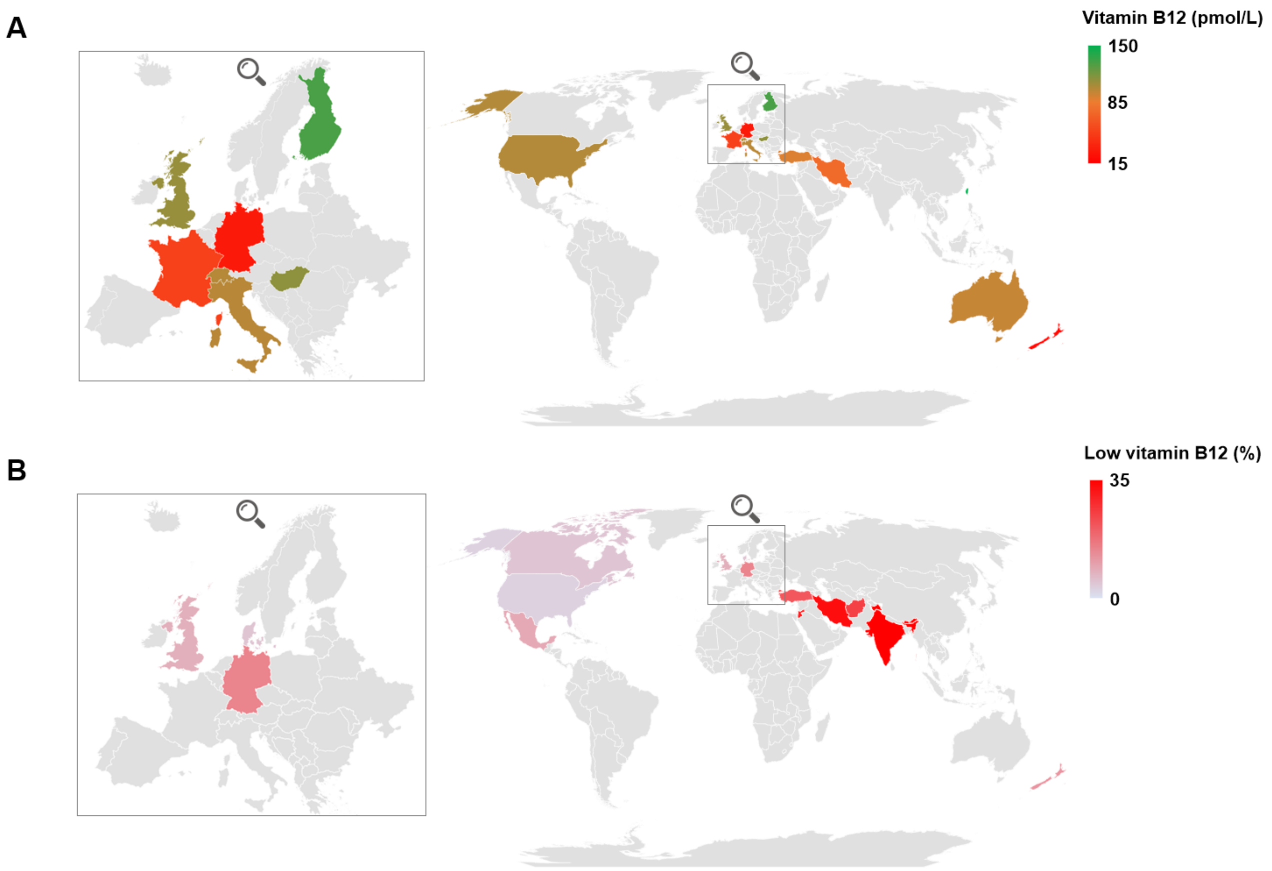

Figure S2. (a) Worldwide landscape of serum or plasma vitamin B12 concentrations among patients with N_2O-induced toxicity. (b) Worldwide prevalence of subjects with low serum or plasma vitamin B12 concentrations among apparently healthy individuals. Prevalence data were extracted from different studies reported around the world [133-142] and from a recently published literature review [143].

References

1. Adornato, B.T. Nitrous oxide and vitamin B12. *Lancet (London, England)* **1978**, *2*, 1318.
2. Burman, J.F.; Amess, J.A.; Mollin, D.L. Nitrous oxide and vitamin B12. *Lancet (London, England)* **1978**, *2*, 1153–1154.
3. Nunn, J.F.; Chanarin, I. Nitrous oxide and vitamin B12. *British journal of anaesthesia* **1978**, *50*, 1089–1090.
4. Chanarin, I. Nitrous oxide and the cobalamins. *Clinical science (London, England : 1979)* **1980**, *59*, 151–154.
5. Chanarin, I. Cobalamins and nitrous oxide: a review. *Journal of clinical pathology* **1980**, *33*, 909–916.
6. Nevins, M.A. Neuropathy after nitrous oxide abuse. *Jama* **1980**, *244*, 2264.
7. Gillman, M.A. Safety of nitrous oxide. *Lancet (London, England)* **1982**, *2*, 1397.
8. Lunsford, J.M.; Wynn, M.H.; Kwan, W.H. Nitrous oxide-induced myeloneuropathy. *J Foot Surg* **1983**, *22*, 222–225.
9. Koblin, D.D.; Biebuyck, J.F. Is nitrous oxide a dangerous anesthetic for vitamin B12-deficient subjects? *Jama* **1986**, *256*, 716.
10. Ueland, P.M. [Nitrous oxide--not only for fun. Nitrogen oxide, vitamin B12 and folate metabolism]. *Tidsskrift for den Norske laegeforening : tidsskrift for praktisk medicin, ny raekke* **1987**, *107*, 2305–2308.
11. Rupreht, J.; Erdmann, W.; Dzoljic, M.; van Stolk, M.A. [Nitrous oxide in anesthesia--present status of the use of nitrous oxide, risks for patients and personnel and treatment of side effects]. *Anaesthesiologie und Reanimation* **1989**, *14*, 251–259.
12. Ostreicher, D.S. Vitamin B12 supplements as protection against nitrous oxide inhalation. *The New York state dental journal* **1994**, *60*, 47–49.
13. Hoerauf, K.; Koller, C.; Wiesner, G.; Taeger, K.; Hobbhahn, J. [Nitrous oxide exposure of operating room personnel in intubation anesthesia]. *Gesundheitswesen (Bundesverband der Arzte des Offentlichen Gesundheitsdienstes (Germany))* **1995**, *57*, 92–96.
14. King, M.; Coulter, C.; Boyle, R.S.; Whitby, R.M. Neurotoxicity from overuse of nitrous oxide. *The Medical journal of Australia* **1995**, *163*, 50–51.
15. Jongen, J.C.; Koehler, P.J.; Franke, C.L. [Subacute combined degeneration of the spinal cord: easy diagnosis, effective treatment]. *Nederlands tijdschrift voor geneeskunde* **2001**, *145*, 1229–1233.
16. Nestor, P.J.; Stark, R.J. Vitamin B12 myeloneuropathy precipitated by nitrous oxide anaesthesia. *The Medical journal of Australia* **1996**, *165*, 174.
17. Takacs, J. [N2O-induced acute funicular myelosis in latent vitamin B 12 deficiency]. *Anasthesiologie, Intensivmedizin, Notfallmedizin, Schmerztherapie : AINS* **1996**, *31*, 525–528.
18. Lai, N.Y.; Silbert, P.L.; Erber, W.N.; Rijks, C.J. "Nanging": another cause of nitrous oxide neurotoxicity. *The Medical journal of Australia* **1997**, *166*, 166.
19. Mayall, M. Vitamin B12 deficiency and nitrous oxide. *Lancet (London, England)* **1999**, *353*, 1529, doi:10.1016/s0140-6736(05)75143-6.
20. Alarcia, R.; Ara, J.R.; Serrano, M.; Garcia, M.; Latorre, A.M.; Capablo, J.L. [Severe polyneuropathy after using nitrous oxide as an anesthetic. A preventable disease?]. *Revista de neurologia* **1999**, *29*, 36–38.
21. Gothe, C.J.; Petersson, G. [Nitrous oxide and cobalamin deficiency]. *Lakartidningen* **1999**, *96*, 5609.
22. Lindstedt, G. [Nitrous oxide can cause cobalamin deficiency. Vitamin B12 is a simple and cheap remedy]. *Lakartidningen* **1999**, *96*, 4801–4805.

23. Barbosa, L.; Leal, I.; Timoteo, A.T.; Matias, T. [Acute megaloblastic anemia caused by inhalation of nitrous oxide in a patient with multiple autoimmune pathology]. *Acta medica portuguesa* **2000**, *13*, 309–312.
24. Vinciguerra, C.; Chazerain, P.; Olivero de Rubiana, J.P.; Moulonguet, A.; Ziza, J.M. [Postoperative combined medullary sclerosis revealing Biermer's disease: toxic effect of nitrous oxide]. *Revue neurologique* **2000**, *156*, 665–667.
25. Smith, I. Nitrous oxide and vitamin B12. *Archives of disease in childhood* **2001**, *85*, 510.
26. Deleu, D.; Hanssens, Y.; Louon, A. Nitrous oxide-induced cobalamin deficiency. *Archives of neurology* **2001**, *58*, 134–135.
27. England, J.M.; Linnell, J.C. Problems with the serum vitamin B12 assay. *Lancet (London, England)* **1980**, *2*, 1072–1074.
28. van Geffen, G.J.; de Boer, H.D.; Liem, T. [Subacute combined degeneration of the spinal cord due to vitamin B12 deficiency: easy diagnosis, effective therapy]. *Nederlands tijdschrift voor geneeskunde* **2001**, *145*, 2195.
29. Burman, J.F. Nitrous oxide and 5,10-methylenetetrahydrofolate reductase. *The New England journal of medicine* **2003**, *349*, 1479–1480; author reply 1479-1480, doi:10.1056/nejm200310093491516.
30. Erbe, R.W.; Salis, R.J. Severe methylenetetrahydrofolate reductase deficiency, methionine synthase, and nitrous oxide--a cautionary tale. *The New England journal of medicine* **2003**, *349*, 5–6, doi:10.1056/NEJMp030093.
31. Yamada, T.; Hamada, H.; Mochizuki, S.; Sutoh, M.; Tsuji, M.; Kawamoto, M.; Yuge, O. General anesthesia for patient with type III homocystinuria (tetrahydrofolate reductase deficiency). *Journal of clinical anesthesia* **2005**, *17*, 565–567, doi:10.1016/j.jclinane.2005.01.005.
32. Gerges, F.J.; Dalal, A.R.; Robelen, G.T.; Cooper, B.; Bayer, L.A. Anesthesia for cesarean section in a patient with placenta previa and methylenetetrahydrofolate reductase deficiency. *Journal of clinical anesthesia* **2006**, *18*, 455–459, doi:10.1016/j.jclinane.2006.01.006.
33. Cohen Aubart, F.; Sedel, F.; Vicart, S.; Lyon-Caen, O.; Fontaine, B. [Nitric-oxide triggered neurological disorders in subjects with vitamin B12 deficiency]. *Revue neurologique* **2007**, *163*, 362–364.
34. El Otmani, H.; El Moutawakil, B.; Moutaouakil, F.; Gam, I.; Rafai, M.A.; Slassi, I. [Postoperative dementia: toxicity of nitrous oxide]. *L'Encephale* **2007**, *33*, 95–97.
35. Luzardo, G.E.; Karlnoski, R.A.; Williams, B.; Mangar, D.; Camporesi, E.M. Anesthetic management of a parturient with hyperhomocysteinemia. *Anesthesia and analgesia* **2008**, *106*, 1833–1836, doi:10.1213/ane.0b013e3181732655.
36. El Otmani, H.; Moutaouakil, F.; Midafi, N.; Moudden, M.; Gam, I.; Hakim, K.; Fadel, H.; Rafai, M.A.; El Moutawakkil, B.; Slassi, I. [Cobalamin deficiency: neurological aspects in 27 cases]. *Revue neurologique* **2009**, *165*, 263–267, doi:10.1016/j.neurol.2008.10.013.
37. Paul, I.; Reichard, R.R. Subacute combined degeneration mimicking traumatic spinal cord injury. *The American journal of forensic medicine and pathology* **2009**, *30*, 47–48, doi:10.1097/PAF.0b013e318187373b.
38. Asghar, A.; Ali, F.M. Anaesthetic management of a young patient with homocystinuria. *Journal of the College of Physicians and Surgeons--Pakistan : JCPSP* **2012**, *22*, 720–722, doi:11.2012/jcpsp.720722.
39. Chaugny, C.; Simon, J.; Collin-Masson, H.; De Beauchene, M.; Cabral, D.; Fagniez, O.; Veyssier-Belot, C. [Vitamin B12 deficiency due to nitrous oxide use: unrecognized cause of combined spinal cord degeneration]. *La Revue de medecine interne* **2014**, *35*, 328–332, doi:10.1016/j.revmed.2013.04.018.

40. Chin, J.; Forzani, B.; Chowdhury, N.; Lombardo, S.; Rizzo, J.R.; Ragucci, M. Rehabilitation essential in the recovery of multifactorial subacute combined degeneration. *Annals of physical and rehabilitation medicine* **2015**, *58*, 190–192, doi:10.1016/j.rehab.2014.12.005.
41. Liakoni, E.; Liechti, M.E. [Not Available]. *Swiss dental journal* **2015**, *125*, 1099–1104.
42. Goodman, B.P. Metabolic and toxic causes of myelopathy. *Continuum (Minneapolis, Minn.)* **2015**, *21*, 84–99, doi:10.1212/01.CON.0000461086.79241.3b.
43. van Amsterdam, J.; Nabben, T.; van den Brink, W. Recreational nitrous oxide use: Prevalence and risks. *Regulatory toxicology and pharmacology : RTP* **2015**, *73*, 790–796, doi:10.1016/j.yrtph.2015.10.017.
44. Garakani, A.; Jaffe, R.J.; Savla, D.; Welch, A.K.; Protin, C.A.; Bryson, E.O.; McDowell, D.M. Neurologic, psychiatric, and other medical manifestations of nitrous oxide abuse: A systematic review of the case literature. *The American journal on addictions* **2016**, *25*, 358–369, doi:10.1111/ajad.12372.
45. Ingelmo, P.; Wei, A.; Rivera, G. Nitrous oxide for procedural analgesia at home in a child with epidermolysis bullosa. *Paediatric anaesthesia* **2017**, *27*, 776–778, doi:10.1111/pan.13150.
46. Fluegge, K. Propionic acid metabolism, ASD, and vitamin B12: Is there a role for environmental nitrous oxide? *International journal of developmental neuroscience : the official journal of the International Society for Developmental Neuroscience* **2017**, *57*, 21–23, doi:10.1016/j.ijdevneu.2016.12.007.
47. Stockton, L.; Simonsen, C.; Seago, S. Nitrous oxide-induced vitamin B12 deficiency. *Proceedings (Baylor University. Medical Center)* **2017**, *30*, 171–172.
48. Layzer, R.B. Myeloneuropathy after prolonged exposure to nitrous oxide. *Lancet (London, England)* **1978**, *2*, 1227–1230.
49. Layzer, R.B.; Fishman, R.A.; Schafer, J.A. Neuropathy following abuse of nitrous oxide. *Neurology* **1978**, *28*, 504–506.
50. Sahenk, Z.; Mendell, J.R.; Couri, D.; Nachtman, J. Polyneuropathy from inhalation of N2O cartridges through a whipped-cream dispenser. *Neurology* **1978**, *28*, 485–487.
51. Gutmann, L.; Farrell, B.; Crosby, T.W.; Johnsen, D. Nitrous oxide-induced myelopathy-neuropathy: potential for chronic misuse by dentists. *J Am Dent Assoc* **1979**, *98*, 58–59.
52. Paulson, G.W. "Recreational" misuse of nitrous oxide. *J Am Dent Assoc* **1979**, *98*, 410–411.
53. Nunn, J.F.; Sharer, N.M.; Gorchein, A.; Jones, J.A.; Wickramasinghe, S.N. Megaloblastic haemopoiesis after multiple short-term exposure to nitrous oxide. *Lancet (London, England)* **1982**, *1*, 1379–1381.
54. Blanco, G.; Peters, H.A. Myeloneuropathy and macrocytosis associated with nitrous oxide abuse. *Archives of neurology* **1983**, *40*, 416–418.
55. Murdoch, B.D.; Fleming, J.H. Pattern-reversal visual evoked potential in a case of nitrous oxide abuse and recovery. *Clin Electroencephalogr* **1985**, *16*, 143–148.
56. Heyer, E.J.; Simpson, D.M.; Bodis-Wollner, I.; Diamond, S.P. Nitrous oxide: clinical and electrophysiologic investigation of neurologic complications. *Neurology* **1986**, *36*, 1618–1622.
57. Schilling, R.F. Is nitrous oxide a dangerous anesthetic for vitamin B12-deficient subjects? *Jama* **1986**, *255*, 1605–1606.
58. Berger, J.J.; Modell, J.H.; Sypert, G.W. Megaloblastic anemia and brief exposure to nitrous oxide--a causal relationship. *Anesthesia and analgesia* **1988**, *67*, 197–198.
59. Stabler, S.P.; Allen, R.H.; Barrett, R.E.; Savage, D.G.; Lindenbaum, J. Cerebrospinal fluid methylmalonic acid levels in normal subjects and patients with cobalamin deficiency. *Neurology* **1991**, *41*, 1627–1632.
60. Vishnubhakat, S.M.; Beresford, H.R. Reversible myeloneuropathy of nitrous oxide abuse: serial electrophysiological studies. *Muscle & nerve* **1991**, *14*, 22–26, doi:10.1002/mus.880140105.

61. Stacy, C.B.; Di Rocco, A.; Gould, R.J. Methionine in the treatment of nitrous-oxide-induced neuropathy and myeloneuropathy. *Journal of neurology* **1992**, *239*, 401–403.
62. Hadzic, A.; Glab, K.; Sanborn, K.V.; Thys, D.M. Severe neurologic deficit after nitrous oxide anesthesia. *Anesthesiology* **1995**, *83*, 863–866.
63. Kinsella, L.J.; Green, R. 'Anesthesia paresthetica': nitrous oxide-induced cobalamin deficiency. *Neurology* **1995**, *45*, 1608–1610.
64. McMorrow, A.M.; Adams, R.J.; Rubenstein, M.N. Combined system disease after nitrous oxide anesthesia: a case report. *Neurology* **1995**, *45*, 1224–1225.
65. Rosener, M.; Dichgans, J. Severe combined degeneration of the spinal cord after nitrous oxide anaesthesia in a vegetarian. *Journal of neurology, neurosurgery, and psychiatry* **1996**, *60*, 354.
66. Brett, A. Myeloneuropathy from whipped cream bulbs presenting as conversion disorder. *Aust N Z J Psychiatry* **1997**, *31*, 131–132, doi:10.3109/00048679709073810.
67. Beltramello, A.; Puppini, G.; Cerini, R.; El-Dalati, G.; Manfredi, M.; Roncolato, G.; Idone, D.; De Togni, L.; Turazzini, M. Subacute combined degeneration of the spinal cord after nitrous oxide anaesthesia: role of magnetic resonance imaging. *Journal of neurology, neurosurgery, and psychiatry* **1998**, *64*, 563–564.
68. Pema, P.J.; Horak, H.A.; Wyatt, R.H. Myelopathy caused by nitrous oxide toxicity. *AJNR. American journal of neuroradiology* **1998**, *19*, 894–896.
69. Jameson, M.; Roberts, S.; Anderson, N.E.; Thompson, P. Nitrous oxide-induced vitamin B(12) deficiency. *Journal of clinical neuroscience : official journal of the Neurosurgical Society of Australasia* **1999**, *6*, 164–166.
70. Lee, P.; Smith, I.; Piesowicz, A.; Brenton, D. Spastic paraparesis after anaesthesia. *Lancet (London, England)* **1999**, *353*, 554, doi:10.1016/s0140-6736(98)10090-9.
71. Ogundipe, O.; Pearson, M.W.; Slater, N.G.; Adepegba, T.; Westerdale, N. Sickle cell disease and nitrous oxide-induced neuropathy. *Clinical and laboratory haematology* **1999**, *21*, 409–412.
72. Sesso, R.M.; Iunes, Y.; Melo, A.C. Myeloneuropathy following nitrous oxide anesthaesia in a patient with macrocytic anaemia. *Neuroradiology* **1999**, *41*, 588–590.
73. Butzkueven, H.; King, J.O. Nitrous oxide myelopathy in an abuser of whipped cream bulbs. *Journal of clinical neuroscience : official journal of the Neurosurgical Society of Australasia* **2000**, *7*, 73–75, doi:10.1054/jocn.1998.0149.
74. Felmet, K.; Robins, B.; Tilford, D.; Hayflick, S.J. Acute neurologic decompensation in an infant with cobalamin deficiency exposed to nitrous oxide. *The Journal of pediatrics* **2000**, *137*, 427–428, doi:10.1067/mpd.2000.107387.
75. Marie, R.M.; Le Biez, E.; Busson, P.; Schaeffer, S.; Boiteau, L.; Dupuy, B.; Viader, F. Nitrous oxide anesthesia-associated myelopathy. *Archives of neurology* **2000**, *57*, 380–382.
76. McNeely, J.K.; Buczulinski, B.; Rosner, D.R. Severe neurological impairment in an infant after nitrous oxide anesthesia. *Anesthesiology* **2000**, *93*, 1549–1550.
77. Iwata, K.; O'Keefe, G.B.; Karanas, A. Neurologic problems associated with chronic nitrous oxide abuse in a non-healthcare worker. *The American journal of the medical sciences* **2001**, *322*, 173–174.
78. Ilniczky, S.; Jelencsik, I.; Kenez, J.; Szirmai, I. MR findings in subacute combined degeneration of the spinal cord caused by nitrous oxide anaesthesia--two cases. *European journal of neurology* **2002**, *9*, 101–104.
79. Ng, J.; Frith, R. Nanging. *Lancet (London, England)* **2002**, *360*, 384, doi:10.1016/s0140-6736(02)09611-3.

80. Selzer, R.R.; Rosenblatt, D.S.; Laxova, R.; Hogan, K. Adverse effect of nitrous oxide in a child with 5,10-methylenetetrahydrofolate reductase deficiency. *The New England journal of medicine* **2003**, *349*, 45–50, doi:10.1056/NEJMoa021867.
81. Waclawik, A.J.; Luzzio, C.C.; Juhasz-Pocsine, K.; Hamilton, V. Myeloneuropathy from nitrous oxide abuse: unusually high methylmalonic acid and homocysteine levels. *WMJ : official publication of the State Medical Society of Wisconsin* **2003**, *102*, 43–45.
82. Diamond, A.L.; Diamond, R.; Freedman, S.M.; Thomas, F.P. "Whippets"-induced cobalamin deficiency manifesting as cervical myelopathy. *Journal of neuroimaging : official journal of the American Society of Neuroimaging* **2004**, *14*, 277–280, doi:10.1177/1051228404264956.
83. Doran, M.; Rassam, S.S.; Jones, L.M.; Underhill, S. Toxicity after intermittent inhalation of nitrous oxide for analgesia. *BMJ (Clinical research ed.)* **2004**, *328*, 1364–1365, doi:10.1136/bmj.328.7452.1364.
84. Miller, M.A.; Martinez, V.; McCarthy, R.; Patel, M.M. Nitrous oxide "whippit" abuse presenting as clinical B12 deficiency and ataxia. *The American journal of emergency medicine* **2004**, *22*, 124.
85. Ahn, S.C.; Brown, A.W. Cobalamin deficiency and subacute combined degeneration after nitrous oxide anesthesia: a case report. *Archives of physical medicine and rehabilitation* **2005**, *86*, 150–153.
86. Waters, M.F.; Kang, G.A.; Mazziotta, J.C.; DeGiorgio, C.M. Nitrous oxide inhalation as a cause of cervical myelopathy. *Acta Neurol Scand* **2005**, *112*, 270–272, doi:10.1111/j.1600-0404.2005.00473.x.
87. Sethi, N.K.; Mullin, P.; Torgovnick, J.; Capasso, G. Nitrous oxide "whippit" abuse presenting with cobalamin responsive psychosis. *Journal of medical toxicology : official journal of the American College of Medical Toxicology* **2006**, *2*, 71–74.
88. Cartner, M.; Sinnott, M.; Silburn, P. Paralysis caused by "nagging". *The Medical journal of Australia* **2007**, *187*, 366–367.
89. Levine, J.; Chengappa, K.N. Exposure to nitrous oxide may be associated with high homocysteine plasma levels and a risk for clinical depression. *Journal of clinical psychopharmacology* **2007**, *27*, 238–239, doi:10.1097/01.jcp.0000264982.02239.48.
90. Shulman, R.M.; Geraghty, T.J.; Tadros, M. A case of unusual substance abuse causing myeloneuropathy. *Spinal Cord* **2007**, *45*, 314–317, doi:10.1038/sj.sc.3101962.
91. Wu, M.S.; Hsu, Y.D.; Lin, J.C.; Chen, S.C.; Lee, J.T. Spinal myoclonus in subacute combined degeneration caused by nitrous oxide intoxication. *Acta neurologica Taiwanica* **2007**, *16*, 102–105.
92. Meyers, L.E.; Judge, B.S. Myeloneuropathy in a dentist. *Clinical toxicology (Philadelphia, Pa.)* **2008**, *46*, 1095–1096, doi:10.1080/15563650802356617.
93. Singer, M.A.; Lazaridis, C.; Nations, S.P.; Wolfe, G.I. Reversible nitrous oxide-induced myeloneuropathy with pernicious anemia: case report and literature review. *Muscle & nerve* **2008**, *37*, 125–129, doi:10.1002/mus.20840.
94. Huang, M.Y.; Tsai, W.; Chang, W.H. Nitrous oxide-induced polyneuropathy in a teenager. *Emergency medicine journal : EMJ* **2009**, *26*, 186, doi:10.1136/emj.2007.057471.
95. Renard, D.; Dutray, A.; Remy, A.; Castelnovo, G.; Labauge, P. Subacute combined degeneration of the spinal cord caused by nitrous oxide anaesthesia. *Neurological sciences : official journal of the Italian Neurological Society and of the Italian Society of Clinical Neurophysiology* **2009**, *30*, 75–76, doi:10.1007/s10072-009-0013-2.
96. Wijesekera, N.T.; Davagnanam, I.; Miszkiel, K. Subacute combined cord degeneration: a rare complication of nitrous oxide misuse. A case report. *The neuroradiology journal* **2009**, *22*, 194–197, doi:10.1177/197140090902200210.

97. Richardson, P.G. Peripheral neuropathy following nitrous oxide abuse. *Emerg Med Australas* **2010**, *22*, 88–90, doi:10.1111/j.1742-6723.2009.01262.x.

98. Tatum, W.O.; Bui, D.D.; Grant, E.G.; Murtagh, R. Pseudo-guillain-barre syndrome due to "whippet"-induced myeloneuropathy. *Journal of neuroimaging : official journal of the American Society of Neuroimaging* **2010**, *20*, 400–401, doi:10.1111/j.1552-6569.2009.00388.x.

99. Alt, R.S.; Morrissey, R.P.; Gang, M.A.; Hoffman, R.S.; Schaumburg, H.H. Severe myeloneuropathy from acute high-dose nitrous oxide (N2O) abuse. *The Journal of emergency medicine* **2011**, *41*, 378–380, doi:10.1016/j.jemermed.2010.04.020.

100. Hathout, L.; El-Saden, S. Nitrous oxide-induced B(1)(2) deficiency myelopathy: Perspectives on the clinical biochemistry of vitamin B(1)(2). *Journal of the neurological sciences* **2011**, *301*, 1–8, doi:10.1016/j.jns.2010.10.033.

101. Lin, R.J.; Chen, H.F.; Chang, Y.C.; Su, J.J. Subacute combined degeneration caused by nitrous oxide intoxication: case reports. *Acta neurologica Taiwanica* **2011**, *20*, 129–137.

102. Probasco, J.C.; Felling, R.J.; Carson, J.T.; Dorsey, E.R.; Niessen, T.M. Teaching NeuroImages: myelopathy due to B(1)(2) deficiency in long-term colchicine treatment and nitrous oxide misuse. *Neurology* **2011**, *77*, e51, doi:10.1212/WNL.0b013e31822c910f.

103. Ghobrial, G.M.; Dalyai, R.; Flanders, A.E.; Harrop, J. Nitrous oxide myelopathy posing as spinal cord injury. *Journal of neurosurgery. Spine* **2012**, *16*, 489–491, doi:10.3171/2012.2.spine11532.

104. Hsu, C.K.; Chen, Y.Q.; Lung, V.Z.; His, S.C.; Lo, H.C.; Shyu, H.Y. Myelopathy and polyneuropathy caused by nitrous oxide toxicity: a case report. *The American journal of emergency medicine* **2012**, *30*, 1016.e1013-1016, doi:10.1016/j.ajem.2011.05.001.

105. Sotirchos, E.S.; Saidha, S.; Becker, D. Neurological picture. Nitrous oxide-induced myelopathy with inverted V-sign on spinal MRI. *Journal of neurology, neurosurgery, and psychiatry* **2012**, *83*, 915–916, doi:10.1136/jnnp-2012-303105.

106. Cheng, H.M.; Park, J.H.; Hernstadt, D. Subacute combined degeneration of the spinal cord following recreational nitrous oxide use. *BMJ case reports* **2013**, *2013*, doi:10.1136/bcr-2012-008509.

107. Chiang, T.T.; Hung, C.T.; Wang, W.M.; Lee, J.T.; Yang, F.C. Recreational nitrous oxide abuse-induced vitamin B12 deficiency in a patient presenting with hyperpigmentation of the skin. *Case reports in dermatology* **2013**, *5*, 186–191, doi:10.1159/000353623.

108. Gursoy, A.E.; Kolukisa, M.; Babacan-Yildiz, G.; Celebi, A. Subacute Combined Degeneration of the Spinal Cord due to Different Etiologies and Improvement of MRI Findings. *Case reports in neurological medicine* **2013**, *2013*, 159649, doi:10.1155/2013/159649.

109. Safari, A.; Emadi, F.; Jamali, E.; Borhani-Haghighi, A. Clinical and MRI manifestations of nitrous oxide induced vitamin B12 deficiency: A case report. *Iranian journal of neurology* **2013**, *12*, 111–113.

110. Arshi, B.; Shaw, S. Subacute ascending numbness. *Clinical toxicology (Philadelphia, Pa.)* **2014**, *52*, 905–906, doi:10.3109/15563650.2014.953170.

111. Dababneh, H.; Hussain, M.; Guerrero, W.R.; Xu, J.; Morgan, W.; Mocco, J.; Kirmani, J.F. "Whippets"-induced vitamin B12 deficiency and dorsal column degeneration. *Journal of vascular and interventional neurology* **2014**, *7*, 8.

112. Garakani, A.; Welch, A.K.; Jaffe, R.J.; Protin, C.A.; McDowell, D.M. Psychosis and low cyanocobalamin in a patient abusing nitrous oxide and cannabis. *Psychosomatics* **2014**, *55*, 715–719, doi:10.1016/j.psym.2013.11.001.

113. Hu, M.H.; Huang, G.S.; Wu, C.T.; Hung, P.C. Nitrous oxide myelopathy in a pediatric patient. *Pediatric emergency care* **2014**, *30*, 266–267, doi:10.1097/pec.0000000000000110.
114. Rheinboldt, M.; Harper, D.; Parrish, D.; Francis, K.; Blase, J. Nitrous oxide induced myeloneuropathy: a case report. *Emergency radiology* **2014**, *21*, 85–88, doi:10.1007/s10140-013-1152-6.
115. Shwe, Y.; Scelsa, S.N. A 23-year-old man with acute onset paresthesias and gait ataxia. *Journal of clinical neuromuscular disease* **2014**, *15*, 192–193, doi:10.1097/cnd.0000000000000035.
116. Duque, M.A.; Kresak, J.L.; Falchook, A.; Harris, N.S. Nitrous Oxide Abuse and Vitamin B12 Action in a 20-Year-Old Woman: A Case Report. *Laboratory medicine* **2015**, *46*, 312–315, doi:10.1309/lm0l9havxchf1uqm.
117. Morris, N.; Lynch, K.; Greenberg, S.A. Severe motor neuropathy or neuronopathy due to nitrous oxide toxicity after correction of vitamin B12 deficiency. *Muscle & nerve* **2015**, *51*, 614–616, doi:10.1002/mus.24482.
118. Pugliese, R.S.; Slagle, E.J.; Oettinger, G.R.; Neuburger, K.J.; Ambrose, T.M. Subacute combined degeneration of the spinal cord in a patient abusing nitrous oxide and self-medicating with cyanocobalamin. *American journal of health-system pharmacy : AJHP : official journal of the American Society of Health-System Pharmacists* **2015**, *72*, 952–957, doi:10.2146/ajhp140583.
119. Thompson, A.G.; Leite, M.I.; Lunn, M.P.; Bennett, D.L. Whippits, nitrous oxide and the dangers of legal highs. *Practical neurology* **2015**, *15*, 207–209, doi:10.1136/practneurol-2014-001071.
120. Wolpert, F.; Barath, K.; Brakowski, J.; Renzel, R.; Linnebank, M.; Gantenbein, A.R. Funicular myelosis in a butcher: it was the cream cans. *Case reports in neurological medicine* **2015**, *2015*, 827168, doi:10.1155/2015/827168.
121. Chen, H.J.; Huang, C.S. Nitrous Oxide-induced Subacute Combined Degeneration Presenting with Dystonia and Pseudoathetosis: A Case Report. *Acta neurologica Taiwanica* **2016**, *25*, 50–55.
122. Hirvioja, J.; Joutsa, J.; Wahlsten, P.; Korpela, J. Recurrent paraparesis and death of a patient with 'whippet' abuse. *Oxford medical case reports* **2016**, *2016*, 41–43, doi:10.1093/omcr/omw012.
123. Massey, T.H.; Pickersgill, T.T.; K, J.P. Nitrous oxide misuse and vitamin B12 deficiency. *BMJ case reports* **2016**, *2016*, doi:10.1136/bcr-2016-215728.
124. Sleeman, I.; Wiblin, L.; Burn, D. An unusual cause of falls in a young woman. *The journal of the Royal College of Physicians of Edinburgh* **2016**, *46*, 160–162, doi:10.4997/jrcpe.2016.304.
125. Buizert, A.; Sharma, R.; Koppen, H. When the Laughing Stops: Subacute Combined Spinal Cord Degeneration Caused by Laughing Gas Use. *Journal of addiction medicine* **2017**, *11*, 235–236, doi:10.1097/adm.0000000000000295.
126. Kaski, D.; Kumar, P.; Murphy, E.; Warner, T.T. Iatrogenic B12-deficient peripheral neuropathy following nitrous oxide administration for functional tonic leg spasm: A case report. *Clinical neurology and neurosurgery* **2017**, *160*, 108–110, doi:10.1016/j.clineuro.2017.07.006.
127. Yuan, J.L.; Wang, S.K.; Jiang, T.; Hu, W.L. Nitrous oxide induced subacute combined degeneration with longitudinally extensive myelopathy with inverted V-sign on spinal MRI: a case report and literature review. *BMC Neurol* **2017**, *17*, 222, doi:10.1186/s12883-017-0990-3.
128. Chen, T.; Zhong, N.; Jiang, H.; Zhao, M.; Chen, Z.; Sun, H. Neuropsychiatric Symptoms Induced by Large Doses of Nitrous Oxide Inhalation: A Case Report. *Shanghai Arch Psychiatry* **2018**, *30*, 56–59, doi:10.11919/j.issn.1002-0829.217084.

129. Egan, W.; Steinberg, E.; Rose, J. Vitamin B12 deficiency-induced neuropathy secondary to prolonged recreational use of nitrous oxide. *The American journal of emergency medicine* **2018**, 10.1016/j.ajem.2018.05.029, doi:10.1016/j.ajem.2018.05.029.

130. Johnson, K.; Mikhail, P.; Kim, M.G.; Bosco, A.; Huynh, W. Recreational nitrous oxide-associated neurotoxicity. *Journal of neurology, neurosurgery, and psychiatry* **2018**, *89*, 897–898, doi:10.1136/jnnp-2017-317768.

131. Keddie, S.; Adams, A.; Kelso, A.R.C.; Turner, B.; Schmierer, K.; Gnanapavan, S.; Malaspina, A.; Giovannoni, G.; Basnett, I.; Noyce, A.J. No laughing matter: subacute degeneration of the spinal cord due to nitrous oxide inhalation. *Journal of neurology* **2018**, *265*, 1089–1095, doi:10.1007/s00415-018-8801-3.

132. Middleton, J.A.; Roffers, J.A. Peripheral Neuropathy Due to Recreational Use of Nitrous Oxide Presenting After an Ankle Sprain With Foot Drop. *Orthopedics* **2018**, *41*, e432-e433, doi:10.3928/01477447-20171102-05.

133. Clarke, R.; Grimley Evans, J.; Schneede, J.; Nexo, E.; Bates, C.; Fletcher, A.; Prentice, A.; Johnston, C.; Ueland, P.M.; Refsum, H., et al. Vitamin B12 and folate deficiency in later life. *Age Ageing* **2004**, *33*, 34–41.

134. McLean, E.; de Benoist, B.; Allen, L.H. Review of the magnitude of folate and vitamin B12 deficiencies worldwide. *Food Nutr Bull* **2008**, *29*, S38-51, doi:10.1177/15648265080292S107.

135. Thuesen, B.H.; Husemoen, L.L.; Ovesen, L.; Jorgensen, T.; Fenger, M.; Linneberg, A. Lifestyle and genetic determinants of folate and vitamin B12 levels in a general adult population. *The British journal of nutrition* **2010**, *103*, 1195–1204, doi:10.1017/S0007114509992947.

136. MacFarlane, A.J.; Greene-Finestone, L.S.; Shi, Y. Vitamin B-12 and homocysteine status in a folate-replete population: results from the Canadian Health Measures Survey. *The American journal of clinical nutrition* **2011**, *94*, 1079–1087, doi:10.3945/ajcn.111.020230.

137. Benson, J.; Phillips, C.; Kay, M.; Webber, M.T.; Ratcliff, A.J.; Correa-Velez, I.; Lorimer, M.F. Low vitamin B12 levels among newly-arrived refugees from Bhutan, Iran and Afghanistan: a multicentre Australian study. *PLoS One* **2013**, *8*, e57998, doi:10.1371/journal.pone.0057998.

138. El-Khateeb, M.; Khader, Y.; Batieha, A.; Jaddou, H.; Hyassat, D.; Belbisi, A.; Ajlouni, K. Vitamin B12 deficiency in Jordan: a population-based study. *Ann Nutr Metab* **2014**, *64*, 101–105, doi:10.1159/000355440.

139. Brito, A.; Mujica-Coopman, M.F.; Lopez de Romana, D.; Cori, H.; Allen, L.H. Folate and Vitamin B12 Status in Latin America and the Caribbean: An Update. *Food Nutr Bull* **2015**, *36*, S109-118, doi:10.1177/0379572115585772.

140. Karabulut, A.; Guler, O.T.; Karahan, H.T.; Ozkan, S.; Koyuncu, H.; Demirciler, I. Premarital screening of 466 Mediterranean women for serum ferritin, vitamin B12, and folate concentrations. *Turk J Med Sci* **2015**, *45*, 358–363.

141. Quay, T.A.; Schroder, T.H.; Jeruszka-Bielak, M.; Li, W.; Devlin, A.M.; Barr, S.I.; Lamers, Y. High prevalence of suboptimal vitamin B12 status in young adult women of South Asian and European ethnicity. *Appl Physiol Nutr Metab* **2015**, *40*, 1279–1286, doi:10.1139/apnm-2015-0200.

142. Sivaprasad, M.; Shalini, T.; Balakrishna, N.; Sudarshan, M.; Lopamudra, P.; Suryanarayana, P.; Arlappa, N.; Ravikumar, B.P.; Radhika, M.S.; Reddy, G.B. Status of Vitamin B12 and Folate among the Urban Adult Population in South India. *Ann Nutr Metab* **2016**, *68*, 94–102, doi:10.1159/000442677.

143. Green, R.; Allen, L.H.; Bjorke-Monsen, A.L.; Brito, A.; Gueant, J.L.; Miller, J.W.; Molloy, A.M.; Nexo, E.; Stabler, S.; Toh, B.H., et al. Vitamin B12 deficiency. *Nature reviews. Disease primers* **2017**, *3*, 17040, doi:10.1038/nrdp.2017.40.

© 2019 by the authors. Submitted for possible open access publication under the terms and conditions of the Creative Commons Attribution (CC BY) license (http://creativecommons.org/licenses/by/4.0/).
